# The completion of indicated paternal prenatal genetic and carrier testing at a public hospital in Los Angeles, California

**DOI:** 10.1016/j.gimo.2023.100831

**Published:** 2023-09-01

**Authors:** Michelle T. Nguyen, Genevieve Mazza, Brian T. Nguyen

**Affiliations:** 1 Division of Maternal-Fetal Medicine, Department of Obstetrics and Gynecology, Keck School of Medicine of the University of Southern California, Los Angeles, CA; 2 Division of Family Planning, Department of Obstetrics and Gynecology, Keck School of Medicine of the University of Southern California, Los Angeles, CA

**Keywords:** Carrier screening, Genetic counseling, Genetic testing, Insurance, Reproductive partner

## Abstract

**Purpose:**

Pregnant individuals are routinely advised to undergo genetic carrier screening, followed by carrier screening in the reproductive partner if the patient’s screen is positive. The objective of our study was to identify completion rates of and barriers to partner carrier screening or genetic testing.

**Methods:**

We conducted a retrospective cohort study examining the completion of indicated partner genetic screening or testing at the Los Angeles General Medical Center Genetics clinic from January 1, 2017, to October 31, 2022. We examined factors linked to completing partner genetic screening or testing, including sociodemographic factors for patients and their partners, testing indications, and pregnancy characteristics via bivariate analyses (eg, *t* test, χ^2^, and Fisher’s exact tests).

**Results:**

In this primarily low-income, publicly insured, Spanish-speaking population, we identified 98 pregnancies for which partner genetic screening or testing was indicated. Only 26.5% (*n* = 26) completed testing, which did not vary by indication, parental age, parental race, parity, or primary language. Completion of partner genetic screening or testing was significantly linked to earlier gestational age at referral for genetic counseling (19.1 versus 21.5 weeks, *P* = .006). In 4 cases, being unable to obtain partner test results led to invasive diagnostic testing of the pregnant patient.

**Conclusion:**

Less than one-third of pregnancies received indicated partner genetic screening or testing. Early referral to genetic counseling may improve partner testing completion rates, which could avoid invasive and unnecessary diagnostic testing in the pregnant patient.

## Introduction

In routine obstetric care, there are certain circumstances in which testing is advised for reproductive partners of pregnant patients. For example, the American College of Obstetricians and Gynecologists (ACOG) recommends universal carrier screening for all pregnant individuals, which includes testing for hemoglobinopathies, cystic fibrosis, spinal muscular atrophy, and Fragile X Syndrome; if a pregnant individual is identified as a heterozygote for any of the autosomal recessive conditions on the carrier screen, both ACOG and the American College of Medical Genetics (ACMG) recommend carrier screening for the reproductive partner to facilitate a more accurate and complete evaluation of the couple’s joint reproductive risks.^1^^,^^2^ Antigen genotyping is, similarly, recommended for partners of pregnant individuals who test positive for an antibody associated with risk of hemolytic disease of the fetus and newborn (HDFN).^3^ Additionally, when a couple has a child with an aneuploidy that could result from nondisjunction, such as Trisomy 21, karyotyping is recommended for both parents to determine the probability of recurrence in subsequent pregnancies.^4^

Despite recommendations from ACOG and ACMG, partner genetic screening or testing is not reliably covered by pregnant patients’ insurance plans, and previous studies note the completion of indicated partner testing in as few as 25.8% of cases.^5^, ^6^, ^7^, ^8^, ^9^ Positive carrier screens in pregnant individuals without follow-up testing in their partners have limited clinical utility and translate to inefficient health care expenditures. More concerning, the reproductive partner’s unknown genetic status puts pregnant individuals and their pregnancies at risk when they decide to undergo invasive diagnostic testing (eg, amniocentesis and cordocentesis) as a consequence of incomplete genetic information about their partner. Identifying and removing barriers to partner genetic screening or testing ensures that pregnancy care is not only complete and cost-effective but also inclusive, reinforcing the significance of partner involvement in pregnancy and childbirth.

This study examines the proportion of reproductive partners who obtained genetic screening or testing (eg, carrier screening, hemoglobin electrophoresis, antigen genotyping, or karyotyping) when clinically indicated and advised by a genetic counselor, with specific attention to factors linked to partner testing completion and the incidence of additional invasive testing in the pregnant patient due to incomplete partner testing at our site.

## Materials and Methods

We conducted a retrospective cohort study at the Los Angeles General Medical Center, examining records from January 1, 2017, to October 31, 2022. The cohort included pregnant patients seen in the Los Angeles General Medical Center Genetics clinic, who received a recommendation from a genetic counselor to obtain partner genetic screening or testing. Indications for partner genetic screening or testing included pregnant patient’s carrier screen positive, pregnant patient’s antibody screen positive, pregnant patient with hemoglobinopathy or thalassemia, pregnant patient with other genetic disorder, abnormal fetal karyotype or microarray, family history of aneuploidy or autism, and prior pregnancy affected by fetal anomaly and/or fetal demise. Of note, carrier screening for all pregnant patients at our institution included spinal muscular atrophy, cystic fibrosis, Fragile X (if family history positive for intellectual disability or autism), and hemoglobinopathies (via electrophoresis if clinically indicated by the patient’s race/ethnicity, hemoglobin level, and/or mean corpuscular volume). Expanded carrier screening was not routinely offered and was not performed unless the patient previously had an expanded carrier screen done at an outside clinic. To obtain partner genetic screening or testing, the partners’ health insurance information was processed to determine whether their testing could be performed in the Los Angeles General Medical Center Genetics clinic on the same day, whether they would need to wait for financial clearance before returning for testing, or whether they would need to obtain testing from an outside provider.

We reviewed the electronic medical records of eligible patients for partner testing indication, completion of partner testing, and reasons for uncompleted partner testing. We defined “completed testing” as testing in which official documentation of the lab results were available for review by the genetic counselor; testing was “incomplete” if solely by self-report or if unable to be verified with documentation. We also collected the following sociodemographic, reproductive, and pregnancy characteristics: parental age, primary language, race/ethnicity (self-identified by the patient on a standardized intake form), gestational age at the time of counseling, parents’ relationship status, pregnant patient’s insurance type, number of living children from the reproductive partner, and the presence of ultrasound anomalies at the time of genetic counseling. We attempted to collect information regarding partner’s insurance for each study participant; however, given its inconsistent documentation in the electronic medical record, we excluded this potential covariate in our analysis. As a secondary objective, we characterized cases in which uncompleted partner genetic screening or testing led to invasive diagnostic testing in the pregnant patient.

We summarized the proportion of patients completing partner genetic screening or testing with simple descriptive statistics and conducted bivariate analyses examining factors linked to completed testing, using *t* tests, χ^2^, and Fisher’s exact tests as appropriate. All data were collected and stored on our institution’s instance of REDCap, a web-based, HIPAA compliant platform for data entry and management.^10^ All statistical analyses were performed using Stata Version 14.2 (StataCorp LLC, College Station, TX). This study was approved by University of Southern California Institutional Review Board.

## Results

We reviewed the records of 98 pregnant patients with indicated partner genetic screening or testing. Their mean gestational age at the time of genetic counseling was 20.9 ± 4.1 weeks; 82.7% received genetic counseling prior to 24 weeks gestation. Most patients (62.2%) identified as Latinx, and the vast majority (93.9%) had state-funded MediCal/Medicaid insurance (Table 1). Over one-third of partner genetic screening or testing recommendations were for positive antibodies in the pregnant patient (37.8%), followed by hemoglobinopathy or thalassemia in the pregnant patient (28.1%) and a prior pregnancy affected by anomalies or fetal demise (15.5%). 97.4% of the 39 patients with a positive antibody screen had at least 1 antibody that can cause severe HDFN, with anti-Kell (*n* = 14) and anti-E (*n* = 12) being the most common antibodies detected (Table 2).^3^^,^^11^^,^^12^

**Table 1:** Completion of partner genetic testing by sociodemographic factors

Baseline Characteristic	Total	Testing Completed	Testing Not Completed	*P* value
	*N* = 98	*n* = 26 (26.5%)	*n* = 72 (73.5%)	
Pregnant patient’s age (mean ± SD, years)				
<25	17 (17.35%)	2 (7.69%)	15 (20.83%)	.239
25-29	26 (26.53%)	6 (23.08%)	20 (27.78%)	
30-34	19 (19.39%)	8 (30.77%)	11 (15.28%)	
35-99	36 (36.73%)	10 (38.46%)	26 (36.11%)	
*Total*	*98 (100%)*	*26 (100%)*	*72 (100%)*	
Gravidity (mean ± SD)				
1	17 (17.35%)	7 (26.92%)	10 (13.89%)	.318
2	20 (20.41%)	4 (15.38%)	16 (22.22%)	
3+	61 (62.24%)	15 (57.69%)	46 (63.89%)	
*Total*	*98 (100%)*	*26 (100%)*	*72 (100%)*	
Parity (mean ± SD)				
0	30 (30.61%)	10 (38.46%)	20 (27.78%)	.937
1	25 (25.51%)	6 (23.08%)	19 (26.39%)	
2	23 (23.47%)	6 (23.08%)	17 (23.61%)	
3+	20 (20.41%)	4 (15.38%)	16 (22.22%)	
*Total*	*98 (100%)*	*26 (100%)*	*72 (100%)*	
Partner’s age (mean ± SD, years)				
<25	8 (9.09%)	1 (4.00%)	7 (11.11%)	.576
25-29	16 (18.18%)	5 (20.00%)	11 (17.46%)	
30-34	18 (20.45%)	7 (28.00%)	11 (17.46%)	
35-99	46 (52.27%)	12 (48.00%)	34 (53.97%)	
*Total*^a^	*88 (100%)*	*25 (100%)*	*63 (100%)*	
Pregnant patient’s race/ethnicity^b^				
White	1 (1.02%)	0 (0.00%)	1 (1.39%)	.695
Latinx/Hispanic	61 (62.24%)	18 (69.2%)	43 (59.7%)	
African American/Black	19 (19.4%)	3 (11.54%)	16 (22.2%)	
Asian	12 (12.24%)	3 (11.54%)	9 (12.50%)	
Other or Unknown	5 (5.10%)	2 (7.69%)	3 (4.17%)	
*Total*	*98 (100%)*	*26 (100%)*	*72 (100%)*	
Partner’s race/ethnicity^b^				
White	1 (1.02)	0 (0.00)	1 (1.39)	.966
Latinx/Hispanic	51 (52.04)	15 (57.69)	36 (50.00)	
African American/Black	19 (19.39)	4 (15.38)	15 (20.83)	
Asian	12 (12.24)	3 (11.54)	9 (12.50)	
Other or Unknown	13 (13.27)	4 (15.38)	9 (12.50)	
*Total*	*98 (100)*	*26 (100)*	*72 (100)*	
Pregnant patient's primary language				
English	63 (64.29)	15 (57.69)	48 (66.67)	.328
Spanish	29 (29.59)	8 (30.77)	21 (29.17)	
Chinese	5 (5.10)	3 (11.54)	2 (2.78)	
Other	1 (1.02)	0 (0.00)	1 (1.39)	
*Total*	*98 (100)*	*26 (100)*	*72 (100)*	
Pregnant patient’s insurance				
None	1 (1.02)	0 (0.00)	1 (1.39)	.252
MediCal or Medicaid	92 (93.88)	23 (88.46)	69 (95.83)	
HMO	2 (2.04)	1 (3.85)	1 (1.39)	
Other or Unknown	3 (3.06)	2 (7.69)	1 (1.39)	
*Total*	*98 (100)*	*26 (100)*	*72 (100)*	
Parents’ relationship status				
No Relationship	5 (5.10)	0 (0.00)	5 (6.94)	.572
In a Relationship but not Married	54 (55.10)	14 (53.85)	40 (55.56)	
Married	35 (35.71)	11 (42.31)	24 (33.33)	
Separated or divorced	4 (4.08)	1 (3.85)	3 (4.17)	
*Total*	*98 (100)*	*26 (100)*	*72 (100)*	
# of living children from current partner				
0	49 (50.00)	12 (46.15)	37 (51.39)	.794
1	22 (22.45)	7 (26.92)	15 (20.83)	
2+	27 (27.55)	7 (26.92)	20 (27.78)	
*Total*	*98 (100)*	*26 (100)*	*72 (100)*	
Gestational age at counseling (mean ± SD, weeks)	20.9 ± 4.13 (CI 20.1, 21.7)	19.15 ± 3.13 (CI 17.9, 20.4)	21.51 ± 4.29 (CI 20.5, 22.5)	.006
Ultrasound-confirmed fetal anomalies				
Yes	7 (7.14)	1 (3.85)	6 (8.33)	.671
No	91 (92.86)	25 (96.15)	66 (91.67)	
*Total*	*98 (100)*	*26 (100)*	*72 (100)*	
Indication for genetic testing^c^				
Pregnant patient’s carrier screen positive	10 (9.71)	2 (7.41)	8 (10.53)	.581
Pregnant patient’s antibody screen positive	39 (37.86)	12 (44.44)	27 (35.52)	
Hemoglobinopathy/thalassemia in pregnant patient	29 (28.16)	9 (33.33)	20 (26.32)	
Other genetic disorder in pregnant patient	4 (3.88)	1 (3.70)	3 (3.95)	
Prior pregnancy with fetal anomaly or demise	16 (15.53)	2 (7.41)	14 (18.42)	
Family history	3 (2.91)	0 (0.00)	3 (3.95)	
Abnormal karyotype or microarray	2 (1.94)	1 (3.70)	1 (1.32)	
*Total*	*103 (100)*	*27 (100)*	*76 (100)*	

a Partner’s age was not reported for 10 patients.

b Racial and ethnic subgroup variations may not be appropriately represented by these data, for which the categories were pre-populated in the electronic medical records.

c If a patient had more than 1 indication for paternal genetic testing, all indications were included. Therefore, the total number of indications exceeds the total number of study subjects.

**Table 2:** Red blood cell antibodies detected on pregnant patients’ antibody screens and their association with HDFN

Antigen	Hemolytic Disease Severity	# of Patients with Antibody
Kell	Mild to severe	14
C	Mild to severe	5
c	Mild to severe	2
D	Mild to severe	3
E	Mild to severe	12
e	Mild	3
Fy^a^	Mild to severe	2
Fy^b^	Mild	1
Jk^a^	Mild to severe	2
Jk^b^	Mild	1
M	Mild to severe	2
Mur	Mild to severe	2
Di^b^	Mild to severe	1
Ge3	Mild to moderate	1
HrB	Severe	1

Approximately one-quarter of patients (26.5%) had partners who completed indicated genetic screening or testing. Completion neither varied by indication for testing (*P* = .58), nor other sociodemographic factors, including age, gravidity, parity, race/ethnicity, primary language, insurance, or relationship status (Table 1). However, partner genetic screening or testing completion was statistically significantly associated with earlier gestational age at time of genetic counseling (19.2 ± 3.1 weeks versus 21.5 ± 4.3 weeks, *P* = .006) (Table 1).

Reasons for uncompleted partner genetic screening or testing could be abstracted from nearly 20% of the records, with the following reasons noted: the partner could not obtain official documentation of test results from an outside clinic (*n* = 6), financial barriers, including lack of insurance or high out-of-pocket costs (*n* = 3), the partner did not want to return to Genetics clinic at a later date for testing (*n* = 2), the partner did not want to schedule a separate appointment with an outside primary care physician (PCP) (*n* = 4), the PCP ordered the incorrect test (*n* = 2), and the partner was deployed outside the country (*n* = 1) or incarcerated (*n* = 1).

We identified 4 cases in which the pregnant patient underwent potentially avoidable, invasive diagnostic testing because of the partner’s inability to obtain genetic screening or testing (Table 3). In 1 case, a pregnant patient with β-thalassemia could not obtain the partner’s verified hemoglobin electrophoresis results from an outside clinic and subsequently underwent amniocentesis to evaluate for fetal thalassemia. In another case, a pregnant patient with a positive antibody screen and elevated peak systolic velocity of the fetal middle cerebral artery underwent cordocentesis because the partner’s red blood cell antigen genotyping was not covered by the partner’s insurance. The fetus turned out to be negative for the antigen and therefore not at risk of alloimmunization.

**Table 3:** Description of cases in which partner genetic testing was not completed, and the pregnant patient underwent invasive diagnostic testing

Case #	Indication for Partner Testing	Reason for Not Completing Partner Testing	Invasive Diagnostic Testing Performed	Indication for Invasive Diagnostic Testing	Relation of Invasive Testing to Insurance Barriers?
1	Positive antibody screen in pregnant patient	Partner’s insurance did not cover red blood cell antigen genotyping	Cordocentesis	Positive antibody screen in pregnant patient with abnormal fetal dopplers	Yes
2	Thalassemia in pregnant patient	Partner could not obtain hemoglobin electrophoresis results from outside clinic	Amniocentesis	Thalassemia in pregnant patient	Yes
3	Abnormal fetal microarray	Partner was incarcerated	Amniocentesis	Ultrasound anomalies	No
4	Positive antibody screen in pregnant patient	Unknown	Amniocentesis	Advanced maternal age	No

## Discussion

ACOG and ACMG endorse genetic screening or testing for reproductive partners when pregnant patients receive a positive screening result. In addition to the professional obligation to follow these guidelines, clinicians have various ethical obligations to provide partner genetic screening or testing, including the obligation to protect pregnant individuals from harmful and potentially unnecessary tests and procedures, the obligation to practice according to evidence-based standards of care, and the obligation to reduce unnecessary costs for the health care system.^13^ Despite these professional endorsements and ethical obligations, 4 previous studies reported partner genetic screening or testing completion rates of less than 50% (Table 4).^5^^,^^6^^,^^8^^,^^9^ In failing to consider reproductive partners’ access to and utilization of partner genetic screening or testing, our health care systems fail to meet these obligations.

**Table 4:** Summary of published research examining the completion of partner carrier testing in pregnancy

Prior Study	Country	*n*	% Partner Testing	Population Characteristics
Carlotti et al (2021)^5^	USA	58	41.4%	Majority White, privately insured pregnant patients; majority of partners had insurance. Timing of genetic counseling and carrier screening (ie, preconception vs prenatal) not reported. Pregnant patient’s insurance type, whether the partner was insured, relationship status, and knowledge of carrier screen were associated with completion of partner carrier screening.
Giles Choates et al (2020)^6^	USA	661	41.5%	Race/ethnicity and insurance information not reported. Highest rate of partner testing completion occurred during preconception and for nulliparous patients; partner testing completion decreased with advancing GA.
Simone et al (2020)^7^	USA	513	77%	Included both nonpregnant and pregnant (*n* = 505) patients. Majority White, privately insured. Most common reasons for incomplete partner testing were pregnant patients lost to follow-up on their own carrier screen results (42%) and belief that partner testing results would not affect pregnancy outcome (20%). GA of pregnant patients not reported.
Arjunan et al (2021)^8^	USA	314,000	25.8% (sequential) 100% (tandem) 95.9% (tandem reflex)	Included both nonpregnant and pregnant (*n* = 134,389) majority White patients. GA of pregnant patients and insurance information not reported. Tandem testing of couples resulted in highest partner testing completion rate (100%) but also a 42.2% rate of unnecessary partner testing. Tandem reflex testing resulted in <1% of unnecessary partner testing. Did not collect reasons for noncompliance in the sequentially screened group.
Shi et al (2021)^9^	China	254	41.2%	Primarily Chinese patients. Average GA at the time of pregnant patient’s carrier screening: 12 weeks. Sequential screening of partners was “self-financed.” Most common reason for declining partner sequential screening was the partner’s previous receipt of normal MCV and MCH on routine blood test, demonstrating low risk of being heterozygous for the thalassemia variant. 8.2% of partners declined screening due to “other” reasons, including high cost.
Nguyen et al^13^^a^	USA	98	26.5%	Primarily low-income, LatinX population at a public hospital. Average GA at time of genetics referral: 21 weeks.

*n*, sample size or number of pregnant people with positive carrier status; *GA*, gestational age.

a Data from current study.

Our study specifically addressed partner genetic screening or testing completion among a publicly insured, primarily Spanish-speaking patient population for which additional structural barriers might further prevent testing completion. The partner testing rate at our institution during a 5-year span was 26.5%. In contrast, Carlotti et al and Simone et al observed partner testing completion rates of 41.4% and 77%, respectively, in their majority White, privately insured patients.^5^^,^^7^ When Carlotti et al stratified their results by demographic variables, they also noted lower rates of partner testing among pregnant individuals who identified as non-White and relied upon Medicaid public insurance.^5^ These patterns of racial and socioeconomic disparities in partner testing completion may reflect problems with access to health care and health literacy, but they may also represent a form of structural racism and implicit bias because prior studies have demonstrated racial bias in genetic counseling and the negative impact of that bias on communication during counseling.^14^, ^15^, ^16^ Studies focusing specifically on Latinx patients note suboptimal knowledge of prenatal genetic testing among these patients even after they received genetic counseling^17^ and a significantly lower acceptance rate of prenatal diagnostic testing among Latinx patients than White and Black patients.^18^ However, as comprehension and recall of genetic testing information among Latinx patients are improved when counseling is delivered in a culturally and linguistically concordant manner,^17^ improvements in the rate of completed testing may be possible with the appropriate allocation of training and resources.

Factors other than race/ethnicity also influence partner testing completion. Despite having a demographically different study population (ie, majority White) compared with our study population, Arjunan et al found a similarly low partner carrier screening rate of 25.8%, specifically among the subset of patients who underwent sequential screening as opposed to tandem or tandem reflex screening.^8^ Based on the findings of Arjunan et al, tandem reflex screening should be offered to reproductive couples to avoid the risk of noncompliance associated with sequential screening while also minimizing the risk of unnecessary partner testing associated with tandem carrier screening.^8^

Offering genetic counseling at an earlier gestational age is another potential strategy to improve partner testing completion. Our study identified a statistically significant difference in completion of partner genetic testing between patients who received genetic counseling at earlier vs later gestational ages. This association was similarly identified in another study of 661 pregnant heterozygotes for a pathogenic variant, in which 17.9% of preconception patients vs 13.4% of first-trimester patients vs 3.5% of second-trimester patients vs 2.8% of third-trimester patients completed partner carrier screening; the overall partner carrier screening completion rate was 41.5%.^6^ Gestational age at the time of entry to prenatal care likely also plays a role because 1 study found that genetic counseling was less likely to be provided to patients who presented for prenatal care at a later gestational age.^19^ Although yet to be studied, the ability to act on a positive test result and intervene at an earlier gestational age when pregnancy termination is still an option may influence genetic counseling referrals and partner genetic screening or testing completion. This is plausible considering that Simone et al found that most reproductive partners who declined testing did so because of the belief that partner testing results would not affect pregnancy outcomes.^7^ In our population, the average gestational age at receipt of counseling was 21 weeks gestation, with 82.7% of our sample receiving counseling before 24 weeks gestation. This leads to an important question of whether state laws regarding access to abortion services and the gestational age cutoff for legally permissible abortions will influence how prenatal genetic testing, including partner testing, is discussed and whether it will be completed.^20^ For example, abortion may not be an option or may not be discussed with patients if they do not receive genetic counseling well before state-determined gestational age limits for abortion. Additional studies, particularly conducted in states with differing gestational age-based abortion restrictions, will be needed to effectively explore this topic.

Out of all the reasons for incomplete partner testing in our study, an inability to obtain documentation of test results from an outside clinic and not wanting to schedule a separate appointment with an outside PCP were the 2 most common reasons for incomplete partner testing. Of the 6 patients in our study who were categorized as having “incomplete” partner testing because of lack of documentation or verification of test results, all 6 reportedly knew their partners’ results (but 1 of the 6 patients had a second indication for partner testing, which was not completed). If the 5 patients with unverified test results who reportedly completed all indicated partner testing were re-categorized as “complete” in our study, the testing completion rate would change only slightly from 26.5% to 31.6%. The requirement for patients to provide verification of test results may be perceived as a barrier to care because patients are then burdened with the responsibility of navigating the health care system to obtain documentation; however, there could be clinical consequences if patients based their medical decision making on self-reported results that turned out to be inaccurate or incomplete, and patients with unverified results should be counseled on this possibility. Different patients accept different levels of uncertainty and risk, and some patients may prefer to treat an unverified result as equivalent to no result, as was the case for 1 of our patients who chose to undergo amniocentesis because she had only verbal but not written information regarding her partner’s carrier status.

Establishing and/or seeking care with non-pregnancy involved primary care providers who are unfamiliar with the recommended testing and unable to effectively justify it to insurance carriers create additional barriers. As a prior study found that reproductive partners who attended the pregnant patient’s initial carrier screening appointment were twice as likely to complete partner carrier screening,^21^ health care systems could leverage the expertise of obstetrician-gynecologists to manage indicated follow-up carrier testing in reproductive partners. Models where clinicians provide sexual and reproductive health care services, such as expedited treatment of sexually transmitted infections, for partners of index patients are already endorsed by the Centers for Disease Control and Prevention and ACOG.^22^ Preliminary studies note their adoption among obstetrician-gynecologists in similar low-resource settings to overcome social and structural barriers to health care commonly observed in male patient populations.^23^^,^^24^

Although prenatal cell-free DNA screening (cfDNA) is becoming increasingly available as an option to screen fetuses for single-gene disorders, red blood cell antigens related to HDFN, and copy number variants (thereby circumventing the need for partner testing), cfDNA is not yet fully validated or approved for indications other than screening for specific chromosomal aneuploidies and fetal D antigen.^25^^,^^26^ In fact, ACOG states that “single-gene cfDNA is not currently recommended in pregnancy” because “there have not been sufficient data to provide information regarding accuracy and positive and negative predictive value in the general population.”^25^ Patients may receive insurance authorization for invasive diagnostic testing but not for cfDNA screening for single-gene disorders and red blood cell antigens, especially if they are publicly insured. Beyond highlighting the low proportion of reproductive partners completing indicated carrier testing, our study specifically described cases in which incomplete testing led to invasive diagnostic testing in the pregnant individual (Table 3). For pregnant individuals who choose to undergo invasive diagnostic testing, amniocentesis is associated with a 0.1% to 0.3% risk of pregnancy loss in addition to risks of harm to the pregnant patient.^27^ Cordocentesis is even more invasive with higher risks of harm, including fetal bleeding, fetal bradycardia, umbilical cord hematoma, infection, and demise.^28^ When partner testing information is inaccessible or unavailable, pregnant patients shoulder the burdens of reproductive health care, which reinforce gender-based inequities. With public insurance coverage and the availability of partner genetic testing, the absorption of these rare but avoidable risks by pregnant individuals is avoidable.

Unlike other studies that observed higher partner testing completion rates among patients who were nulliparous,^6^ married,^5^ or privately insured,^5^ our study did not show an association between partner testing completion and parity, relationship status, or pregnant patients’ insurance type. Our study is limited by its modest sample size and the incomplete recording of various patient characteristics (eg, partner’s insurance, parental education level, and parental religion), which likely prevented our analysis from observing associations of partner carrier testing completion with previously described patient characteristics. Because of sample size limitations, we also could not stratify the results by type of carrier screen or pre- vs post-COVID timing to analyze the effects of these variables on partner testing completion. Furthermore, our study only included OB clinic patients who received genetic counseling in our Genetics clinic but did not include other prenatal care patients at Los Angeles General Medical Center who had an indication for partner testing and were never given a referral or appointment for genetic counseling. If we had included these patients, we expect that the rate of partner testing completion would be even lower. We additionally note the potential value of examining the impact of including the reproductive partner in genetic counseling, which we were unable to study because of efforts to reduce face-to-face contact during the COVID-19 pandemic, when genetic counseling was performed solely through telehealth beginning in March 2020 and when reproductive partners may not have been routinely offered the option to attend genetic counseling sessions and obtain testing. We recognize the limited generalizability of these findings to low-income minority populations, although we note that these findings highlight the importance of allocating resources to a group in need of intervention.

### Conclusions

Approximately one-quarter of pregnancies for which partner genetic evaluation was indicated at our institution had testing completed, which highlights ongoing discrepancies between established standards of care and actual practice, particularly in public hospital settings. We identified cases in which incomplete or missing information regarding partner carrier status led to unnecessary diagnostic procedures. Additional studies are needed to further corroborate these findings to identify barriers to partner genetic screening or testing and interventions for reducing these barriers, all of which is needed to ensure the equitable and just care of pregnant individuals.

## Data Availability

The data that support the findings of this study are available on request from the corresponding author. The data are not publicly available because of privacy or ethical restrictions.

## Conflict of Interest

Brian T. Nguyen is on the research advisory boards of Myovant Sciences and Sebela Pharmaceuticals, the products of which are unrelated to this topic.
